# A seesaw of equilibrium, midwives’ experiences of infection prevention and control guideline adherence: A qualitative descriptive study

**DOI:** 10.1177/17571774241245259

**Published:** 2024-04-06

**Authors:** Maura McCarthy, Martina Giltenane, Owen Doody

**Affiliations:** 1 University Maternity Hospital, Limerick, Ireland; 2 Health Research Institute, Department of Nursing and Midwifery, 150229 University of Limerick, Limerick, Ireland

**Keywords:** Infection prevention and control, guidelines, midwives experiences, barriers and facilitators, qualitative descriptive

## Abstract

**Background:**

Infection prevention and control guidelines play a key role in preventing infections which can impact mothers and their newborn’s quality of life. Despite the presence of evidenced-based infection prevention and control guidelines, midwives’ adherence can be suboptimal internationally. The identification of facilitators and barriers to infection prevention and control guidelines can support practice and facilitate midwifery care.

**Aim:**

To understand midwives’ experiences of the barriers and facilitators when adhering to infection prevention and control guidelines.

**Methods:**

A qualitative descriptive study using semi-structured interviews with 10 midwives from February to March 2022. The interviews were audio recorded, transcribed verbatim, and analysed utilising Braun and Clarke’s thematic analysis framework involving the six steps of becoming familiar with the data, generating initial codes, generating themes, reviewing themes, defining and naming the themes, and presenting themes.

**Findings:**

Two themes developed; seesaw for equilibrium and back to basics: learning on your feet. Midwives experienced conflicting emotional motivators in the need for professional integrity towards infection prevention and control guideline adherence. The work environment impacts on midwives’ ability to adhere to guidelines and communication and education have a vital role to play in infection prevention and control guideline adherence.

**Conclusions:**

While midwives have a strong sense of protection of professional integrity, work conditions such as environment, organisational structures, and management systems affect midwives’ adherence to infection prevention and control guidelines. Effective education, training, and communication are required to promote infection prevention and control guideline adherence.

## Introduction

Infections obtained because of healthcare treatment are known as healthcare-associated infections and can be prevented by antimicrobial stewardship and the application of an infection prevention and control (IPC) programme (Friedrich, 2019). Death, long-term illness, and serious acute illness can occur, consequently, there is also a resource cost to healthcare providers in terms of infection treatment such as isolation provisions and extended hospital stays (Health Information and Quality Authority, 2017). Predominantly women choose to have their babies in the hospital setting (Department of Health, 2016) and expect quality care. This includes midwives’ adherence to IPC guidelines (Health Information and Quality Authority, 2016) which form the cornerstone of dealing with and constraining infections in the healthcare system (Atif et al., 2013) and prevention harm to healthcare professionals, healthcare users, and the visiting public (World Health Organization, 2020a).

Clinical guidelines are a statement construct detailing evidence-based recommendations for healthcare decision-makers and clinical staff to ensure safe quality of care (Jemal et al., 2020). The implementation of clinical guidelines promotes the provision of high-quality safe healthcare (Mitchell et al., 2020). IPC guidelines apply scientific evidence to everyday practice in IPC but unfortunately, the adherence of healthcare workers has been seen as suboptimal (Lailawidar et al., 2022). For example, hand hygiene (HH) remains suboptimal despite multiple strategies to improve clinical practices (Srigley et al., 2015). Clinical guidelines contain systematically developed recommendations for clinical practice (Wang et al., 2018) and are intended to support clinical decision-making and improve the quality of care, patient outcome, and cost-effectiveness (Greenhalgh et al., 2014). However, caution regarding clinical guidelines is noted especially where evidence is lacking, misleading, or misinterpreted (Pacini et al., 2016). In addition, guideline development groups often lack the time, resources, and skills to gather and scrutinise the evidence (Holmes et al., 2015). A recent review of the literature (McCarthy et al., 2023) highlights barriers and facilitators for midwives adhering to IPC guidelines. McCarthy et al., (2023) emphasised four aspects, compliance is infrastructure and resource-dependent, midwives recognise and work with what they have, midwives experience fear and anxiety regarding adherence, and culture change is a mammoth challenge. In terms of compliance being infrastructure and resource-dependent, it is evident that economic, infrastructure, and organisational factors affect the provision of adequate resources (Abdalrahman et al., 2018) and health systems came under immense pressure with the onset of the pandemic. In the context of recognising and working with what you have, it is established that factors are beyond the control of the midwife, for example, the quality of personal protective equipment (PPE) (Kabasakal et al., 2021), equipment disinfection and hand hygiene (Nalule et al., 2021) and midwives often make their judgment and personal risk assessment (Bouchoucha and Moore 2018). With regards to midwives' fear and anxiety, it is apparent that the quick and ever-changing guidance developed during the pandemic (Asefa et al., 2022), caused confusion (Abutheraa et al., 2020) and a fear of contracting an infection (Alonso et al., 2021). Regarding culture change being a mammoth challenge it was apparent that those with good hand hygiene practices were less likely to be infected (Atnafie et al., 2021), staff behaviours were a source of the spread of infection (Mitchell et al., 2021), and education is vital to promote adherence to guidelines (Abdalrahman et al., 2018). Overall, McCarthy et al. (2023) review highlights that the studies were not designed to examine facilitators and barriers of midwives' adherence to IPC guidelines and thus this study aims is to explore midwives’ experiences of the barriers and facilitators regards IPC guideline adherence.

## Method

### Design

A qualitative descriptive research design was chosen to capture participants’ experiences, aiming to gather rich descriptive data (Bradshaw et al., 2017; Neergaard et al., 2009). The study adhered to the consolidated criteria for qualitative research reporting (COREQ) (Tong et al., 2007) (see Supplemental file 1).

### Ethical considerations

Ethical approval (REC REF: 133/2021) was obtained from the hospital’s Research Ethics Committee. Participants provided voluntary consent and were informed of their right to withdraw without consequences. Confidentiality was assured through data pseudonymization, storage on password-protected computers, and adherence to data protection guidelines (Department of Justice, 2018).

### Recruitment

Ten midwives from a University Maternity Hospital in Ireland participated in the study. Following access from the Director of Midwifery purposive sampling ensured a range of experience levels among participants who met specific criteria: voluntary participation, registration with the national nursing and midwifery board, at least 1 year of experience, and capacity to provide informed consent. Study details were disseminated through posters and interested individuals received an invitation letter, an informational leaflet, and an informed consent form. Participants returned signed consent forms in provided envelopes. Sample size was determined by data saturation, achieved with 10 participants, ensuring comprehensive coverage of concepts in the transcripts (Malterud et al., 2016).

### Data collection and analysis

A pilot study (*n* = 2) (Doody and Doody, 2015) tested the interview schedule, developed from literature review and team experience. Pilot participants were excluded from the main study. Data collected via audio-recorded semi-structured interviews (February to March 2022) were transcribed verbatim. Interviews lasted approximately 30 min, using open-ended questions and probing. An interview schedule (Table 1) guided discussions; a member check was offered but not requested. Braun and Clarke’s (2021) thematic analysis framework were used for data analysis, involving: (1) becoming familiar with data, (2) generating initial codes, (3) generating themes, (4) reviewing themes, (5) defining and naming themes, and (6) presenting themes.

**Table 1.** Interview guide.

Interview questions
How many years have you been practicing as a midwife?
Have you completed any academic studies related to infection prevention and control?
How prepared were you for your role in infection prevention and control?
What guides you in your practice in relation to infection prevention and control?
Do you feel supported to adhere to the infection prevention and control guidelines?
Has anything influenced you to revisit the infection prevention control guidelines in your area of work?
What facilitates you to implement infection prevention and control guidelines?
What prevents you from implementing infection prevention and control guidelines?
Prompts included
What was your experience?
Can you tell me more about?
You mentioned?
Concluding questions
Is there anything else that you would like to add?

### Rigour and trustworthiness

In qualitative research, rigour ensures the trustworthiness of findings (Colorafi and Evans, 2016; Morse, 2015), achieved through consistent procedural application. Rigour was addressed by purposeful sampling for rich participant descriptions, a pilot study for interview schedule testing, reaching data saturation, offering transcript verification, and confirming coding and findings with participants. Additionally, two authors (MMcC, OD) independently coded and reviewed codes, subthemes, and themes, maintaining a clear audit trail through discussion.

## Results

Ten midwives participated in this study and participant demographics are presented in Table 2. The thematic analysis utilised Braun and Clarke’s thematic framework (Braun and Clarke, 2021) resulting in codes, subthemes, and themes identified in Table 3. Two themes were identified from the data analysis; (1) the seesaw for equilibrium; and (2) back to basics: learning on your feet. Within the results, midwives are referred to as ‘participants’, and service users are referred to as ‘women’.

**Table 2.** Demographic details.

Characteristics	*n* = 10	%
Gender		
Female	10	100
Male	0	0
Mean level of midwifery experience	17.7	
Highest level of professional qualification		
Master’s degree	1	10
Higher diploma	4	40
Bachelor of science degree	4	40
Certificate	1	10
Further professional training		
Infection prevention and control Master’s degree	1	10
Infection prevention and control module	1	10

**Table 3.** Codes, subthemes, and themes.

Codes	Subthemes	Themes
Fear		
Frustration		
Guilt		
Anxiety		
Need for feeling safe	The midwife, internal forces	
Confidence		
Confusion as changing guidelines		
Need to protect patient and self		
Tiredness and decision-making		
Physical effect of PPE		
New norm – changing behaviour		
Wanting to do your best		
Professional integrity and pride in work	Midwifery as a profession	Seesaw for equilibrium
Role models		
Effect of hospital management structure		
Midwives being responsible for all ward activities		
Organisational structures		
Strong leadership		
Management styles		
Reactive not proactive	Organisational structure	
Time to read guidelines		
Need for a supportive environment		
Tic box exercise		
Staffing levels		
Managerial system, not being listened to, causing knock of confidence		
Feeling valued		
		
Knowledge of and value of IPC guidelines	Presence the guidelines	
Making own decisions on guidelines		
Availability of guidelines access		
Leadership		
IT difficulties		
Promoting effective communication		
Communication tools (ISBAR)	Communication	
Introduction of electronic resources (Q-pulse, ICNET)		
Communication barrier (PPE) women		Back to basics: Learning on your feet
Different expectations for education		
Importance of education		
Narrow focus on HH and PPE		
Evidence of CPD		
No CPD	Education	
Educational methods		
Short-term influences for guideline adherence		
Types and quality of information sessions		
Learning versus being told		
Move education to ward setting		
Back to basics		
Undergraduate programme		

### Seesaw for equilibrium

The internal influencing factors that participants experienced when striving to adhere to IPC guidelines were evident in this theme. Participants identified the need to find a balance between the demands and uncertainty of everyday practice and the desire to practice safely. *‘Fear’* and *‘anxiety’* caused participants to seek guidelines and there was a refocus on IPC guidelines due to COVID-19: *‘They [the IPC guidelines] guided us through’ and ‘I really saw the benefit’ (Ciara)*. Participants wanting to *‘protect them [mother and baby]’* and *‘protect ourselves’* and challenges such as guidelines *‘constantly changing’* led to *‘confusion’* and *‘frustration*’. Participants expressed a difficulty in balancing the ability to adhere to the IPC guidelines when doing shift work such as night duty, long working days and the physical demands of the participants’ work affected participants’ ability to adhere to guidelines.

*‘It's hard to remember to do everything right and correctly and follow the measures all the time after a long day or night shift.’* [Cathy].

The participants identified balancing the provision of a safe environment and maintaining professional integrity and standards as important. Participants saw role modelling, through adherence to standards and requirements as imperative for professionalism and the education and training of student midwives to promote best practices for the future.

*‘You have a responsibility to pass on and demonstrate the best practice to the students because they are the future of the profession.’* [Polly].

*‘The manager is coming up and like they've got their long hair, not tied up, they're wearing rings, they have nail Polish on and like that's really not leading by example and you know.’* [Vera].

Dealing with the effect of one’s workload and balancing workloads contributed to behaviours deviating from the IPC guidelines in the clinical practice setting.

‘*You know you're run ragged trying to do your own caseload and to look after women and then trying to figure in all the administrator work that we have and then trying to adhere to your IPC, it’s a lot.’* [Rachel].

Participants identified that there is a need to recalibrate, as accepted norms and behaviours were adopted as part of the delivery of care and become part of the culture, but these can deviate from policy, procedures, and guidelines.

*‘I suppose what happens is that we can’t see the woods from the trees, and you need to separate out what’s in the guidelines and what has just become part of practice in this hospital.’* [Joan].

### Back to basics: Learning on your feet

The importance of teamwork and ‘*support from our colleagues’* was highlighted as important to participants in their daily practice. Participants expressed frustration in having to deal with other disciplines who appeared to adopt a culture of *‘blame the midwife*’ when matters relating to IPC were not put in place correctly even though IPC was deemed important across all disciplines and not just a midwifery concern. Participants identified a need to return to basic role identification and questioned the need for all health professionals to be required to clean a bed space.

*‘Like the cleaning of a bed space or beds, it's like, one person’s job to clean the space then its someone else's job to clean mattress.’* [Rachel].

At an environmental level, the *‘old’* and *‘not fit for purpose’* building infrastructure, physical layout and facilities of wards affected the participant’s ability to adhere to IPC guidelines.

*‘The buildings are very old, and the infrastructure isn't great, so there's not a separate sink to facilities us to wash our hands.’* [Sally].

Within the environment, participants recognised the impact of visual clues like the presence of hand sanitiser units at eye level and posters for handwashing and infection control. However, some participants felt there was an overuse of posters, and the behavioural prompter was lost as they stated there was a ‘*visual overload’* and they ‘*end up not seeing it’*. The introduction of the ‘buddy system’ to support the donning and doffing of PPE was beneficial and the repetition of the donning and doffing leading to a behavioural change and learning in practice.

‘*We've been doing the buddy system and it has helped me to adhere correctly and I suppose it helped us change our behaviour.’* [Jennifer].

Due to the onset of coronavirus participants felt that some of the other guidelines were overshadowed which affected adherence as there was a focus on PPE and HH.

*‘Segregating the linen correctly is just as important and I feel there's little emphasis on this anymore as we have been so focused on COVID.’* [Vera].

The basics of availability of resources including *‘time to read the guidelines’, ‘reusing single-use items’,* and *‘substandard PPE’* was an issue for participants and caused associated issues such as cleaning requirements, role identification, and additional guideline adherence.

*‘There is a lack of equipment, there is a lot of sharing of equipment you know how each patient should have their own and you don’t have the staff and sometimes the equipment.’* [Polly].

Participants saw the value of leadership from the ward manager to encourage and facilitate adherence to guidelines. However, organisational structure and leadership style influenced adherence as there was a feeling of a *‘tick box’* exercise stemming from management rather than valuing and leading adherence. Some participants felt ‘*IT challenged’* them as they reported difficulties in accessing the guidelines and ended up *‘making their own decisions based on experience’*:

*‘Something you would not have access to them and what I do, I don't think about doing it, it's just I just know what to do.’* [Rosie].

Participants reported that PPE caused a communication barrier with the women and impacted the care provided, ‘*it made it hard*’, especially with bereaved parents. Participants had to adjust and use alternative communication strategies and question what level of PPE was appropriate. This led to confusion regarding the wearing of facemasks and there was a lack of support from hospital management on this issue which resulted in feelings of not being listened to and being undervalued. There was a clear need for a *‘supportive environment’* to apply the basic IPC measures, this affected the relationship between the participants and management, and having to go it alone and sort themselves.

*‘We felt like we didn’t matter and weren’t being listened to it was hard.’* [Ciara].

From a service perspective, participants experienced a *‘reactive rather than proactive’* approach from hospital management when there was an IPC issue. If a needle stick injury occurred, there was *‘a big drive’* to make sure the sharps guidelines were read and signed by all staff. However, participants felt there was a missed opportunity as the *‘momentum for change’* lessened before changes could be implemented and participants reverted to old patterns of behaviour again, so little learning occurred. Communication and communication tools in the dissemination of guidelines were highlighted by participants as many felt *‘under-prepared*’.

What was helpful and supported learning were *‘the huddle’* and *‘ISBAR’* (Identify, Situation, Background, Assessment and Recommendation, a mnemonic created to improve safety in the transfer of critical information). However, to meet the basics for practice participants identified the need for an on-site member of the IPC team to monitor behaviours, act as a resource for education, train and promote best practices, and identified ward-based learning as a more effective means of learning, ‘*you’re on the floor and gaining experience*’. For education to translate to learning and into practice participants, felt there was a need to focus on behavioural change with a refocus on the ‘basics’ concerning IPC.

‘*We need more education, but that education needs to focus on our behaviour and now we can change that otherwise, it won’t work, reading or having a talk is not enough to bring it back to practice and then your left to your own knowledge or understanding and applying that to your practice be it right or not.’* [Carmel].

## Discussion

This study emphasises midwives' challenges in balancing daily practice and safety, particularly heightened during the pandemic. Factors such as work patterns, environment, and resource availability impact guideline adherence. Visual cues and teamwork facilitate adherence, but role clarity and visual overload can affect effectiveness. Strategies like the buddy system, huddle, ISBAR, and behaviour-focused education, along with management support, are crucial. However, PPE can hinder communication with women, necessitating support for alternative strategies.

Fear and anxiety regarding infection transmission and the quality and reuse of single-use PPE were prominent concerns for participants in this study, and drive midwives' adherence to IPC guidelines (Abbas and Sultan, 2021; Buxton et al., 2019; Scicluna and Attard, 2021). The evolving landscape of the COVID-19 pandemic and rapidly changing guidelines, coupled with PPE availability issues and inadequate communication, caused stress and confusion among midwives. Clear communication is crucial for guideline dissemination and engagement (MacIntyre and Chughtai, 2020; Semann et al., 2020; Silva et al., 2021). The World Health Organization (2020b) stresses the importance of keeping healthcare professionals informed to prepare them mentally and physically for job demands. Management support is vital in this preparation (Semann et al., 2020); however, participants in this study reported a lack of support, such as initially being denied permission to wear facemasks during the pandemic, leading to added stress, anxiety, and feelings of undervaluation. Wearing PPE, fear for personal and family safety, knowledge burden from rapidly changing guidelines, and inner conflicts regarding demands and needs were identified as sources of stress and anxiety for midwives during the pandemic (Wilson et al., 2021). This fear parallelled experiences during the SARS outbreak (Wong et al., 2005), and the temporary PPE availability and quality issues in this study reflected global shortages due to COVID-19 (Rich-Edwards et al., 2021).

Role clarity regarding resource availability and IPC adherence emerged as a concern among participants in this study, with healthcare workers' understanding of their roles influencing IPC compliance (Shah et al., 2015). Clear identification of roles and responsibilities in IPC management is essential; for instance, promoting IPC through resources like single-use blood pressure cuffs and decontamination efforts (Atnafie et al., 2021; Solomon et al., 2018). Ambiguity in role definitions can lead to varying interpretations and inconsistent practices (Gesser-Edelsburg et al., 2018). Shift work and fatigue were noted to compromise standards, aligning with research showing decreased compliance during long shifts (Dai et al., 2015). Participants in this study expressed a desire to provide optimal care for women and infants despite struggling with the physical demands of midwifery practice. Communication barriers created by PPE further impacted care delivery (Alnuaimi, 2021; Alonso et al., 2021). However, participants commended the midwifery profession’s response during the COVID-19 pandemic (Aksoy and Koçak, 2020). On the other hand, conflicts between IPC measures and traditional training, as well as concerns regarding professional authority, were acknowledged (Raveis et al., 2014). Participants emphasised the importance of role models in influencing IPC behaviours among colleagues and students (Al-Maani et al., 2022; Ward, 2013). Effective role models must adhere to hand hygiene guidelines (Erasmus et al., 2009).

This study revealed how accepted norms and behaviours influence IPC guideline adherence. Healthcare workers often adapt to organisational norms rather than challenging suboptimal practices (Herbeć et al., 2020), resulting in a low prioritisation of IPC (Smiddy et al., 2015). Fostering a safety culture is crucial for enhancing patient outcomes (Mitchell et al., 2020; Powell-Jackson et al., 2020). Leadership and teamwork were found to promote quality care and safe practices (Abutheraa et al., 2020), emphasising the need for proactive managerial responses to IPC guideline adherence (Van Buijtene and Foster, 2019). Developing new norms and behaviours is vital for improving adherence (Gesser-Edelsburg et al., 2021). Participants in this study noted the effectiveness of behavioural patterns resulting from the ‘buddy system’ for donning and doffing PPE, which involves an observer ensuring correct steps are followed (Das and Rajalingham, 2020; Poller et al., 2018). However, behavioural change presents challenges, involving complex social interactions and relations (May et al., 2014). It is influenced by social mechanisms such as coherence, cognitive participation, collective action, and reflexive monitoring (Johnson and May, 2015).

This study highlights the significant demand on staff, with visual cues like posters improving compliance but also leading to visual overload. Participants noted both helpfulness and counterproductivity of such cues due to message saturation (Bouchoucha and Moore 2018; Clack et al., 2019). While education is vital for effective IPC, participants expressed consensus on suboptimal IPC education, emphasising the need for more ward-based education and a permanent IPC champion for guidance and training (Ashinyo et al., 2021; Perera et al., 2022). However, education alone may not suffice to drive behaviour change and implementation, as evidenced by the inadequate PPE training highlighted during the COVID-19 pandemic (Gordon and Thompson 2020; Haegdorens et al., 2022). Though short videos are recommended for training, time constraints and limited access to electronic resources pose challenges (Christensen et al., 2020). Balancing educational demands on midwives is crucial, considering factors like time, experience, and institutional culture (Regmi and Jones, 2020). Examining educational approaches within organisations can identify strategies to overcome implementation barriers and address training needs (Tabak et al., 2017) and future studies should employ implementation science methodologies to ensure consistency and measure educational strategy impact.

Participants noted a narrow focus on certain aspects of standard IPC precautions during the COVID-19 pandemic, leading to neglect of other elements. Suboptimal reuse of PPE components, identified in the study, poses infection risks for midwives and women/babies, potentially resulting in care failures (Kurinczuk et al., 2022; Nguyen et al., 2020). The physical environment was cited as a barrier to IPC guideline adherence, contributing to infection transmission. Input from IPC authorities should inform planning for new healthcare environments and recommendations for existing facilities.

## Limitations

While this study sheds light on barriers and facilitators to IPC guideline adherence, its generalisability is limited by its single-site and relatively small sample. Nonetheless, the insights gained may be applicable across various settings. Social desirability bias may have led some nurse participants to withhold their true opinions, potentially affecting the disclosure of barriers influencing their practice. Additionally, biases such as socially desired responses and self-selection may be present, impacting the representation of the wider group. The interviews' average duration of 30 min, constrained by time availability and work demands, may have restricted participants' expression of thoughts and issues. However, participants were given the opportunity to raise additional points at the end of each interview and could contact the researcher for further contributions.

## Conclusion

Overall, this study highlighted fear, anxiety, professional integrity, and protection as key motivators for guideline adherence. During the COVID-19 pandemic, rapid guideline changes led to confusion and frustration, exacerbated by limited access to guidelines due to computer resource availability and IT skills. The work environment and organisational structures, including management and leadership, and role models influenced adherence. An IPC champion and permanent on-site IPC team member are essential for ward-based training and support, addressing midwives' feelings of unpreparedness. Education plays a crucial role in guideline implementation and as no one single strategy can be linked directly to successful implementation. Future studies should employ implementation science methodologies to ensure consistency in reporting strategies, measurement, and impact.

## Supplemental Material

Supplemental Material - A seesaw of equilibrium, midwives’ experiences of infection prevention and control guideline adherence: A qualitative descriptive study Supplemental Material for A seesaw of equilibrium, midwives’ experiences of infection prevention and control guideline adherence: A qualitative descriptive study by Maura McCarthy, Martina Giltenane and Owen Doody in Journal of Infection Prevention
